# Maternal *vgll4a* regulates zebrafish epiboly through Yap1 activity

**DOI:** 10.3389/fcell.2024.1362695

**Published:** 2024-02-20

**Authors:** Carlos Camacho-Macorra, Noemí Tabanera, Elena Sánchez-Bustamante, Paola Bovolenta, Marcos J. Cardozo

**Affiliations:** ^1^ Centro de Biología Molecular Severo Ochoa, Consejo Superior de Investigaciones Científicas-Universidad Autónoma de Madrid, Madrid, Spain; ^2^ Centro de Investigación Biomédica en Red de Enfermedades Raras (CIBERER), Madrid, Spain

**Keywords:** YAP signaling, VGLL4, vgll4a, actomyosin, E-cadherin complex, epiboly

## Abstract

Gastrulation in zebrafish embryos commences with the morphogenetic rearrangement of blastodermal cells, which undergo a coordinated spreading from the animal pole to wrap around the egg at the vegetal pole. This rearrangement, known as epiboly, relies on the orchestrated activity of maternal transcripts present in the egg, compensating for the gradual activation of the zygotic genome. Epiboly involves the mechano-transducer activity of yap1 but what are the regulators of yap1 activity and whether these are maternally or zygotically derived remain elusive. Our study reveals the crucial role of maternal vgll4a, a proposed Yap1 competitor, during zebrafish epiboly. In embryos lacking maternal/zygotic *vgll4a* (MZ*vgll4a*), the progression of epiboly and blastopore closure is delayed. This delay is associated with the ruffled appearance of the sliding epithelial cells, decreased expression of yap1-downstream targets and transient impairment of the actomyosin ring at the syncytial layer. Our study also shows that, rather than competing with yap1, vgll4a modulates the levels of the E-cadherin/β-catenin adhesion complex at the blastomeres’ plasma membrane and hence their actin cortex distribution. Taking these results together, we propose that maternal *vgll4a* acts at epiboly initiation upstream of yap1 and the E-cadherin/β-catenin adhesion complex, contributing to a proper balance between tissue tension/cohesion and contractility, thereby promoting a timely epiboly progression.

## Introduction

The three-dimensional organization of multicellular organisms requires morphogenetic tissue rearrangements, which are particularly evident during embryonic development or in pathological conditions, such as wound healing or cancer. These rearrangements are largely coordinated by the interplay among cell-cell interaction, mechanical forces, and signaling pathways, derived, in part, from the surrounding environment. The adaptive morphogenetic outcome critically depends on how the cells integrate and interpret this signal complexity and transmit the message to their nuclei, eliciting a consequent transcriptional response (Wozniak and Chen, 2009).

In several organisms, including the teleost zebrafish, epiboly is the first morphogenetic rearrangement of the gastrulating embryo (Warga and Kimmel, 1990). These cellular rearrangements are relatively simple and thus have been used to understand basic principles of morphogenesis (Kimmel et al., 1995; Bruce, 2016). The zebrafish blastoderm, positioned at the animal pole, is formed by a single layer of loosely packed cells, known as deep cell layer (DEL), which is covered by the so called epithelial enveloping layer (EVL). The blastoderm interfaces with the yolk syncytial layer (YSL), itself a derivative of the blastoderm marginal cells. During epiboly, the blastoderm thins and moves towards the vegetal pole together with the external YSL (E-YSL). These epithelial spreading and thinning are driven, in part, by a circumferential actomyosin network formed by the E-YSL at its border with the EVL. The contraction of this actomyosin ring pulls the EVL to surround the yolk, further dragging the DEL toward the vegetal pole. This process culminates at the end of gastrulation with “blastopore closure” when the blastoderm seals around the yolk (Kimmel et al., 1995; Bruce, 2016).

In zebrafish, gastrulation commences during maternal-to-zygotic transition, a pivotal process marked by the activation of the zygotic genome but with a substantial involvement of maternal transcripts/proteins that endure throughout gastrulation, actively participating in its morphogenesis (Solnica-Krezel, 2020). Several studies have shown that teleost epiboly involves the function of the transcriptional co-activator Yes1 Associated Transcriptional Regulator (Yap1) and of the highly related TAZ (Gee et al., 2011; Hu et al., 2013; Porazinski et al., 2015; Vázquez-Marín et al., 2019). Yap1 was first described as a downstream nuclear effector of the Hippo signaling pathway, which is involved in the control of cell proliferation (Zhao et al., 2007). However, there is evidence that Yap1 can operate, perhaps independently of Hippo signaling, as a mechano-transducer that links mechanical forces to cellular response (Dupont et al., 2011). Cells constantly probe the forces generated in their extracellular environment through plasma membrane adhesive proteins and internal tension adjustments executed by modifications of their actomyosin network. Upon increasing intracellular tension, Yap1, which is normally retained in the cytoplasm, translocates to the nucleus, where it binds to TEA domain (TEAD)-containing transcription factors (TEAD1–TEAD4). The derived complex activates the transcription of target genes, which include F-acting regulators and cell adhesion components, thereby promoting a positive feedback loop that enables cells to counterbalance the external mechanical forces to which they are exposed (Panciera et al., 2017). Consistent with such mechano-transducer activity, yap1 has been shown to regulate medaka fish epiboly progression by controlling the expression of *arhgap18*, a gene encoding a Rho GTPase activating protein involved in the contraction of the actomyosin ring at the E-YSL/EVL interface (Porazinski et al., 2015). How yap1 activity is controlled and whether this is exerted by maternally or zygotically derived factors remained unanswered.

Vestigial-like protein 4 (Vgll4) is a transcriptional cofactor that can bind directly to TEAD proteins, *via* its tandem Tondu domain (TDU), thereby competing for the Yap-TEAD interaction and thus mechanistically acting as an inhibitor of Yap activity (Koontz et al., 2013; Jiao et al., 2014; Zhang et al., 2014; Li et al., 2023). In *Xenopus* and zebrafish the transcripts of the different *vgll4* paralogs show a dynamic developmental expression pattern in different tissues (Faucheux et al., 2010; Barrionuevo et al., 2014; Xue et al., 2018) and in zebrafish the mRNA of two (Xue et al., 2018) (*vgll4a*; *vgll4b*) of the three paralogs (*vgll4a*, *vgll4b* and *vgll4l*) have been detected at the 1 cell stage (Xue et al., 2018), suggesting a possible early function. Although there are additional and species-specific phenotypes that involve later developing organs (Fillatre et al., 2019; Xue et al., 2019; Wang et al., 2020), loss of function studies in mice and analysis of genetic variants in the teleost *Scatophagus argus* suggest that Vgll4 participates in the regulation of body size (Feng et al., 2019; Suo et al., 2020; Yang et al., 2020; Sheldon et al., 2022), a trait that is often altered as the consequence of an abnormal gastrulation.

By generating mutants for the three *vgll4* paralogs in zebrafish, here we have explored if vgll4 could be involved in controlling yap1 activity during epiboly. We show that maternal but not zygotic vgll4a is required to sustain yap1 signaling and E-cadherin/β-catenin adhesion complex, thereby promoting cell cortex configuration and actomyosin contraction, which drive epiboly progression and, ultimately, an appropriate elongation of the embryonic antero-posterior axis.

## Results

### *vgll4a* function is required for proper epiboly progression

To determine whether *vgll4* paralogues contribute to zebrafish epiboly, we generated zebrafish lines in which *vgll4a*, *vgll4b* and *vgll4l* genes were mutated using CRISPR-Cas9 technology (Supplementary Figure S1A). Founders carrying frame-shift mutations for either one of the three *vgll4* paralogs were selected in the F1 generation, in order to obtain loss of function lines for each one of the existing Vgll4 proteins (Supplementary Figure S1B). Homozygous mutants for *vgll4a*, *vgll4b* and *vgll4l* genes were obtained in F3. Their offspring were viable with no evident developmental phenotypes (Supplementary Figure S1C and data not shown). Similar results were obtained when adult *vgll4a*;*vgll4b* double mutants were analyzed (Supplementary Figure S1C).

*vgll4a* and *vgll4b* but not *vgll4l* transcripts are maternally expressed (Xue et al., 2018). We thus searched a possible phenotype in the F4 generation, in which *vgll4a* or *vgll4b* parental contribution will be no longer active. By intercrossing adults F3 *vgll4a*, *vgll4b*, *vgll4l* and *vgll4a*;*vgll4b* double mutant, we generated F4 embryos lacking both maternal and zygotic contribution of *vgll4a* (MZ*vgll4a*), *vgll4b* (MZ*vgll4b*), *vgll4l* (MZ*vgll4l*) and *vgll4a;vgll4b* (MZ*vgll4a;*MZ*vgll4b*). The fertilization rate of the obtained heterozygous M*vgll4a* and M*vgll4b* embryos was significantly lower than in wild type (wt) and heterozygous *vgll4a* or *vgll4b* embryos (Supplementary Figures S2A-C). In contrast, the fertilization rate of M*vgll4l* heterozygous was similar to that of wt and heterozygous *vgll4l* embryos (Supplementary Figure S2D). This observation suggests that maternal contribution of both *vgll4a* and *vgll4b* could be important for oogenesis.

**FIGURE 1:**
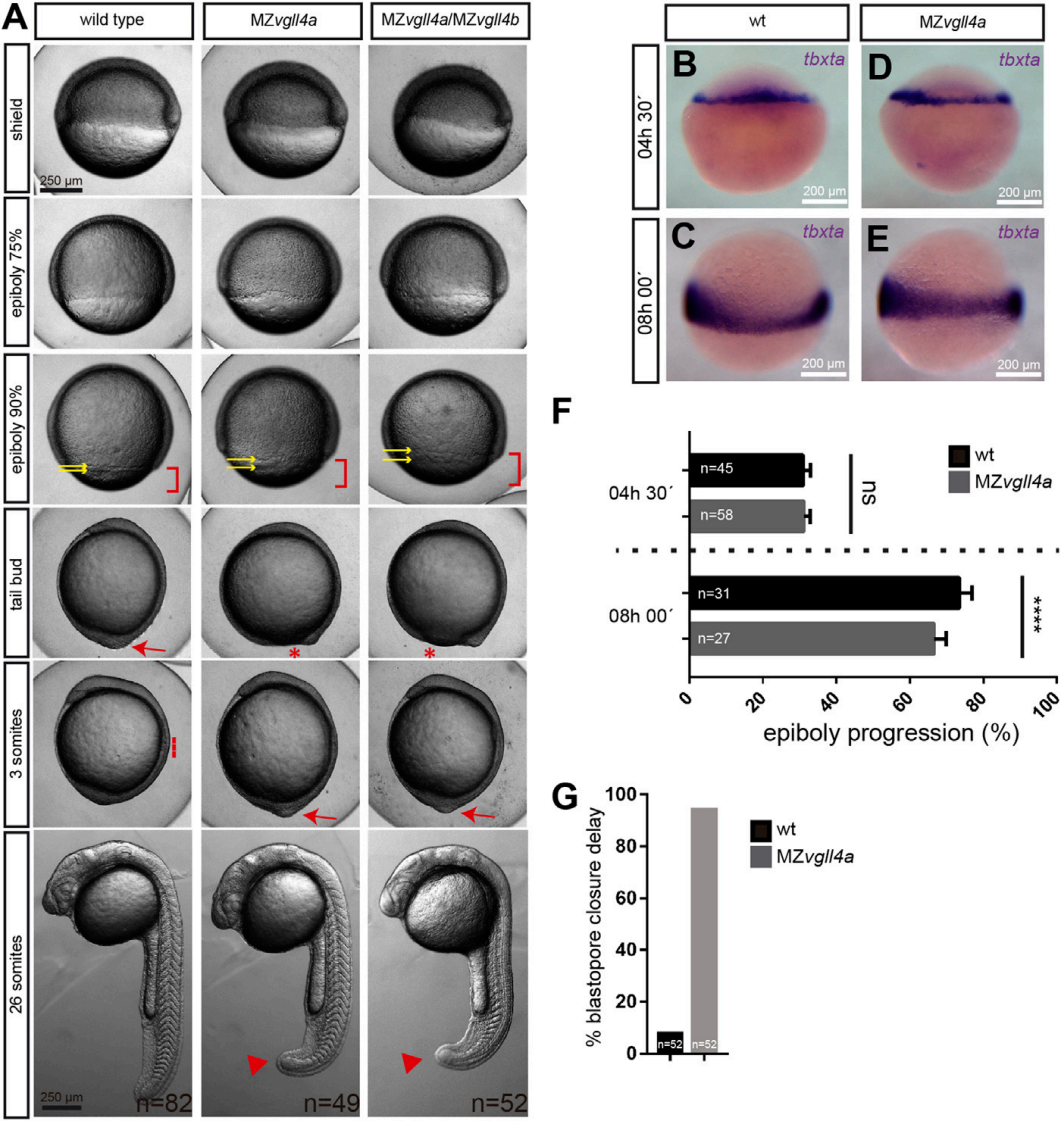
Epiboly progression in MZ*vgll4a* embryos is delayed. **(A)** Bright field images of wt, MZ*vgll4a* and MZ*vgll4a;*MZ*vgll4b* embryos at different developmental stages as indicated in the panels for wt embryos. Note the delayed epiboly (red brackets) and blastopore closure (red asterisks) as well as the increased distance between the EVL and DEL margins (yellow arrows) in MZ*vgll4a* and MZ*vgll4a;*MZ*vgll4b* embryos as compared to wt. Blastopore closure (red arrow) in mutants occurs when wt embryos have initiated somitogenesis (red dotted line). At the 26 somites’ stage, the tail of mutants is still not fully elongated (red arrowheads). **(B–E)** Margin cells in wt **(B,C)** and MZ*vgll4a* **(D,E)** embryos stained by *in situ* hybridization of *tbtxa* at 4.5hpf **(B,D)** and 8hpf **(C,E)**. **(F)** Quantification of epiboly progression of wt and MZ*vgll4a* embryos stained by *in situ* hybridization of *tbtxa* at 4.5hpf and 8hpf. T-test, *p* < 0.0001. **(G)** Quantification of wt and MZ*vgll4a* embryos that do not reach blastopore closure at 10hpf. Fisher’s exact test, *p* < 0.0001.

Despite the decrease in the fertilization rate, viable MZ*vgll4a* embryos appeared to develop normally at least until the 64-cells’ stage (wt, n = 21; MZ*vgll4a,* n = 20 embryos). However, once epiboly begun, their development progressed at a slower pace than in wt embryos (Figure 1A). MZ*vgll4a;*MZ*vgll4b* double mutants were no different from MZ*vgll4a* embryos (Figure 1A), indicating that either maternal *vgll4b* does not contribute to this process or that both paralogs are involved. Comparison of the position of *tbxta-*positive margin cells in wt and MZ*vgll4a* embryos confirmed the beginning and extent of this delay in the mutants (Figures 1B–F), that culminated with a considerable delay in their blastopore closure (Figures 1A, G). This support the idea that *vgll4a* function contributes to epiboly progression.

### The function of *vgll4a* and *vgll4b* is required for larval body lengthening

**FIGURE 2:**
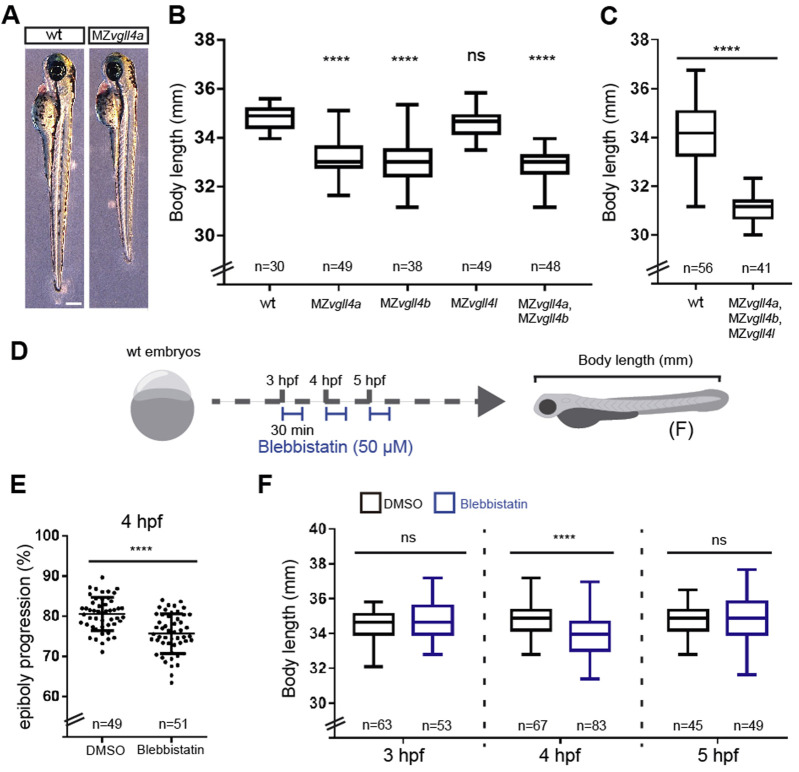
Body length is affected in MZ*vgll4* larvae which could be related to epiboly progression delay. **(A)** Lateral views of wt and MZ*vgll4a* larvae at 3dpf (left) and graph **(B)** showing the quantification of the body length from wt, MZ*vgll4a,* MZ*vgll4b*, MZ*vgll4l* and MZ*vgll4a*;*MZvgll4b* larvae at 3dpf. Note that MZ*vgll4a*, MZ*vgll4b* and MZ*vgll4a*;MZ*vgll4b* but not MZ*vgll4l* mutants are shorter that the wt. Mann Whitney test, *p* < 0.0001. **(C)** The body length of MZ*vgll4a*;*MZvgll4b*;MZ*vgll4l* triple mutant larvae are also shorter than wt at 3dpf. Kruskal–Wallis test, *p* < 0.0001. **(D)** Schematic representation of the experimental design in **(F)**. **(E)** The blebbistatin treatment at 4hpf during 30 min, delays epiboly progression in wt embryos **(F)** The treatment of wt embryos with blebbistatin at 4hpf during 30 min affects the body length of the larvae at 3dpf. Mann Whitney test, *p* < 0.0001. But not at 3hpf nor 5 hpf. Mann Whitney test, *p* = 0.2545 and *p* = 0.4396 respectively. T-test, *p* < 0.0001. Ns, not significant. ****p < 0.0001.

A previous report suggests a relationship between epiboly delay and reduced larvae body size although the precise relationship between the two events has not been fully elucidated (Sun et al., 2017). We thus compared the antero-posterior (A-P) axial length of wt and MZ*vgll4a* larvae at 3dpf, finding a shorter body length in the mutants as compared to the wt (Figures 2A, B). A similar reduced A-P growth was also observed in MZ*vgll4b* and MZ*vgll4a;*MZ*vgll4b* 3dpf larvae but not in the MZ*vgll4l* ones, which presented a size comparable to that of wt (Figure 2B). Notably, complete *vgll4* inactivation in the triple MZ*vgll4a;*MZ*vgll4b;*MZ*vgll4l* mutants, was also followed by a considerably shorter A-P size of the mutant larvae at 3dpf (Figure 2C), ruling out effects derived by possible transcriptional compensations among vgll4 paralogs (Sztal and Stainier, 2020).

To provide evidence that the reduced body size of MZ*vgll4a* larvae is linked to a delay in epiboly progression, we pharmacologically interfered with this process by treating wt embryos with blebbistatin (50 μM), a myosin 2 inhibitor that impairs actin accumulation in the YSL, thereby delaying epibolic movements (Köppen et al., 2006). Notably, a 30 min treatment at 4hpf, was sufficient to delay epiboly progression − as quantified by the position of *tbxta-*positive margin cells (Figure 2E) − and to induce a short axis phenotype when treated animals reached 3dpf larval stage (Figure 2F). Notably, treatments at 3hpf or 5hpf did not result in such a short axis phenotype (Figure 2F).

Together these data support an association between a poor function of myosin 2, epiboly delay and shorter larval size. Nevertheless, factors other than myosin activation are likely contributing to the observed MZ*vgll4a* phenotype, as calyculin-mediated over-activation of myosin did not rescue the body size of the mutant larvae (Supplementary Figure S3).

### Maternal but not zygotic *vgll4a* contribution is necessary for a timely development

Many factors implicated in epiboly are maternally derived (Lepage and Bruce, 2010). The phenotype observed in MZ*vgll4a* embryos was not observed in Z*vgll4a* mutant embryos obtained from heterozygous in-crosses (data not shown), suggesting that maternal but not zygotic *vgll4a* contributes to epiboly progression.

**FIGURE 3:**
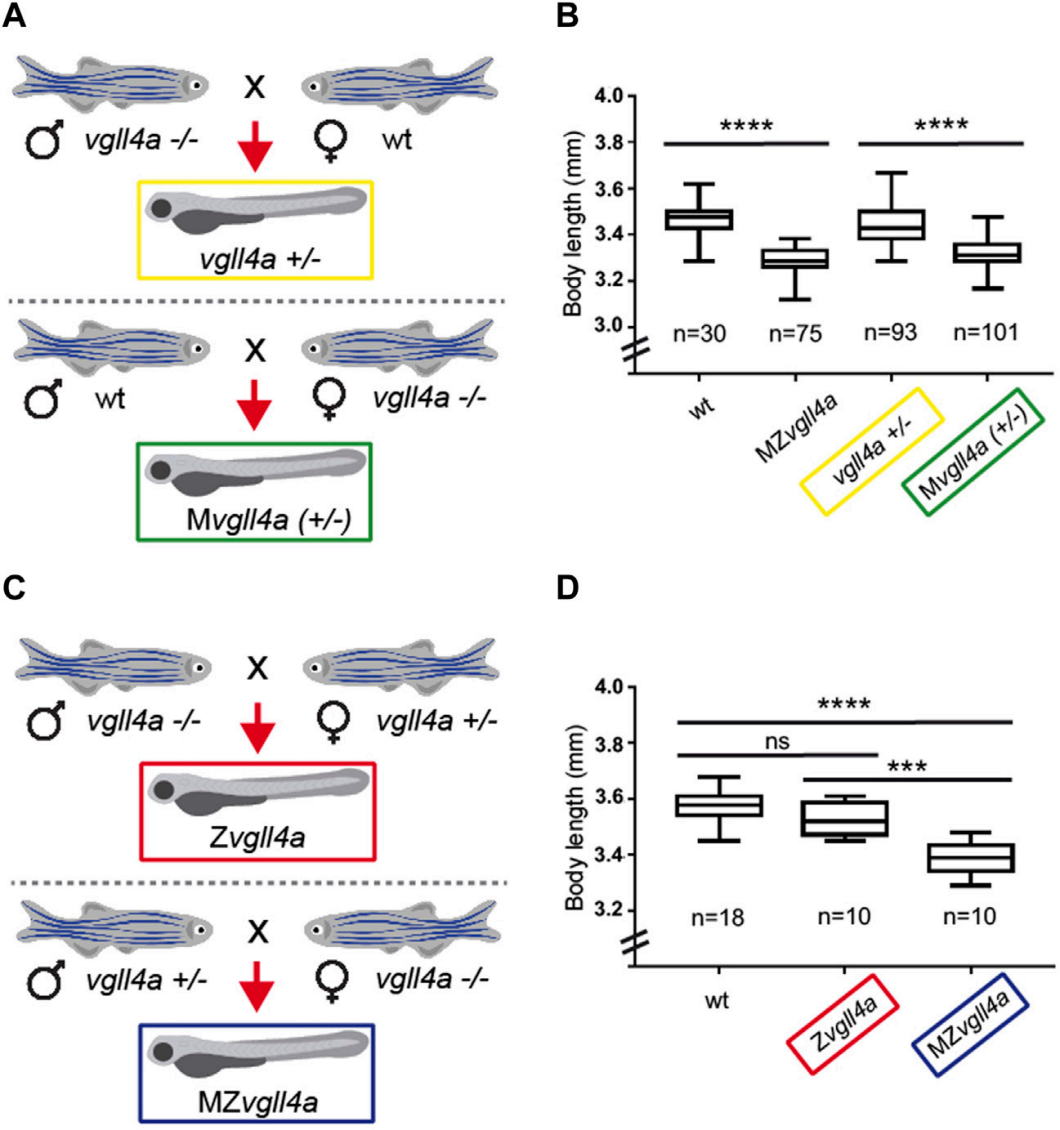
Zygotic *vgll4a* function is dispensable for timely embryonic growth. **(A,C)** Schematic representation of the mating strategy used to obtain embryos of the desired genotypes with or without *vgll4a* maternal contribution. **(B,D)** Box plots of the body length from 3dpf larvae of the indicated genotypes. Note the decreased body length only in embryos with absent or haplo-insufficient maternal *vgll4*a contribution as compared to wt. The number of analyzed embryos is indicated below each plot. Data in B were analyzed with Kruskal–Wallis test, *p* < 0.0001, whereas those in D with One-Way ANOVA. ns, not significant. ***p < 0.001. ****p < 0.0001.

To test this possibility, we mated wt, heterozygous and mutant *vgll4a* fishes to generate larvae of different genotypes with or without *vgll4a* maternal contribution (Figures 3A, C). Determination of the body length of the resulting larvae showed that, similarly to MZ*vgll4a*, M*vgll4a* ^*+/−*^ embryos had an A-P axis shorter than that of *vgll4a* ^*+/−*^ and wt embryos (Figure 3B). This indicates that zygotic *vgll4a* cannot compensate for the lack of its maternal function. To demonstrate that maternal *vgll4a* is indeed sufficient, we crossed *vgll4a* mutant males with *vgll4a* heterozygous females and *vgll4a* heterozygous males with *vgll4a* mutant females to obtain Z*vgll4a* and MZ*vgll4a* embryos, with or without maternal *vgll4a* contribution, respectively (Figure 3C). Notably, the body length of the resulting Z*vgll4a* larvae at 3dpf was very comparable to that of wt (Figure 3D), indicating that the observed developmental delay is linked exclusively to the absence of *vgll4a* maternal contribution.

We next asked if *vgll4b* could have a similar maternally restricted function*.* MZ*vgll4b* were significantly shorter than wt but the body length of M*vgll4b* ^*+/−*^ larvae was comparable to that of wt (Supplementary Figure S4B), indicating that zygotic *vgll4b* compensates for its maternal function and that the MZ*vgll4b* short axis phenotype is exclusively linked to the absence of *vgll4b* zygotic contribution (Supplementary Figure S4D).

To verify that the phenotype observed in MZ*vgll4a* larvae is directly linked to the absence of the maternally inherited mRNA/protein, we sought to rescue MZ*vgll4a* embryonic growth by injecting either the *vgll4a-HA* mRNA, its mutated version or the human VGLL4 protein in 1 cell stage embryos (Supplementary Figure S5A). None of the two mRNAs could rescue the growth of the mutant embryos (Supplementary Figure S5B), despite the presence of the HA-tagged protein in the blastomeres as determined by immunofluorescent staining (Supplementary Figures S5C, D). Injection of recombinant human VGLL4 protein instead partially rescued the growth of MZ*vgll4a* embryos (Supplementary Figure S5E), likely because its immediate availability compensates for the maternal component.

Taken altogether, these observations demonstrate that maternal, but not zygotic, *vgll4a* is essential for timely embryonic development.

### Vgll4a sustains Yap1 signaling to promote embryonic development

Vgll4 competes with Yap1 for TEAD binding in different biological contexts, thereby antagonizing its signaling (Koontz et al., 2013; Jiao et al., 2014; Zhang et al., 2014; Geng et al., 2021; Mickle et al., 2021; Cai et al., 2022). According to this mechanism, the MZ*vgll4a* mutants should present an over-activation of Yap signaling and thus, Yap inhibition should rescue the MZ*vgll4a* phenotype.

Direct testing of this possibility proved to be difficult as Western blot with three antibodies did not detect any of the Yap forms in lysates of 6hpf embryos. Furthermore, our attempt to cross the MZ*vgll4a* line with the Yap/Taz-Tead responsive transgenic reporter line 4xGTIIC:eGFP (Miesfeld and Link, 2014) was unsuccessful, possibly explaining why in the original description positive signal was reported from 22hpf onward (Miesfeld and Link, 2014). To overcome these difficulties, we turned to pharmacological treatments.

**FIGURE 4:**
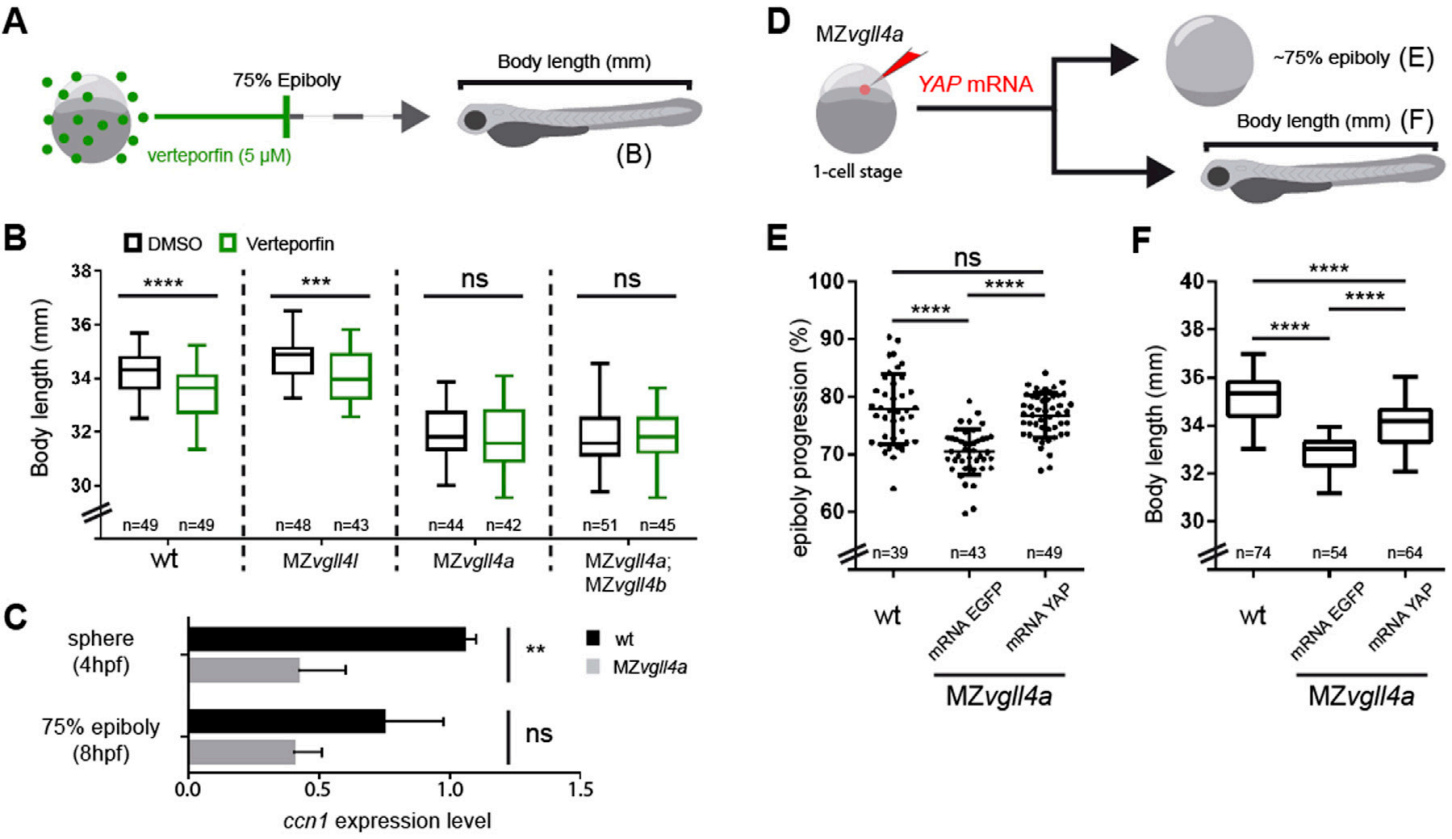
Vgll4a acts upstream of yap activity to promote embryonic growth. **(A)** Schematic representation of the experimental design in **(B)**. **(B)** Box plots of the body length from wt, MZ*vgll4l*, MZ*vgll4a* and MZ*vgll4a;*MZ*vgll4b* embryos grown in the presence of verteporfin or DMSO. Note that the drug has no effect on the already reduced body length of MZ*vgll4a* and MZ*vgll4a;*MZ*vgll4b* larvae but reduces that of wt and *MZvgll4l* larvae. T-test. wt, *p* < 0.0001; MZ*vgll4l, p* = 0.0003; MZ*vgll4a, p* = 0.5769 and MZ*vgll4a;*MZ*vgll4b, p* = 0.9744. **(C)** The graphs show the expression level of the Yap-TEAD transcriptional target *ccn1* at sphere and 75% epiboly stage in wt and *MZvgll4a* embryos as determined by qRT-PCR analysis. Note the significant reduction of *ccn1* expression in the mutants. T-test. At 4hpf, *p* = 0.0042 and at 8hpf, *p* = 0.0746. **(D)** Schematic representation of the experimental design of data reported in **(E,F)**. **(E)** YAP mRNA injection rescues epiboly progression in MZ*vgll4a* embryos to an extend similar to that of wt embryos whereas eGFP mRNA has no effect. Kruskal–Wallis test, *p* < 0.0001. wt vs. MZ*vgll4a* mRNA YAP injected, *p* > 0.9999. **(F)** MZ*vgll4a* embryos injected with YAP mRNA display a larvae body length larger than MZ*vgll4a* embryos injected with control mRNA. One-Way ANOVA, *p* < 0.0001. Ns, not significant. **p < 0.01. ***p < 0.001. ****p < 0.0001.

Verteporfin acts as an efficient inhibitor of YAP-TEAD interaction, reducing Yap1 signaling in different models (Liu-Chittenden et al., 2012), including the zebrafish. We thus soaked normal (wt) and MZvgll4 embryos in this drug using a lower concentration (5uM) and shorter incubation times than those previously reported for older zebrafish embryos (Grampa et al., 2016; Fillatre et al., 2019) in order to prevent potential harmful effects in early stage embryos. We used the timeframe from one-cell stage to 75% epiboly, which is ideal to early maternal vgll4a activity. Embryos were let develop in fresh medium and their body length was measured at 3dpf (Figure 4A), finding that the A-P lengths of wt and MZ*vgll4l* larvae was reduced (Figure 4B) as in MZ*vgll4a* larvae (Figure 2B). Verteporfin instead had no effect on the A-P length of MZ*vgll4a* and MZ*vgll4a;*MZ*vgll4b* larvae, which were undistinguishable from their respective untreated mates (Figure 4B). This suggests that in this scenario, vgll4a and yap1 functions do not compete one another but rather act in the same direction. To verify this possibility, we asked if yap signaling was decreased in MZ*vgll4a* embryos, using the expression levels of the yap1 targets *ccn1* and *ccn2* as read-outs (Zhang et al., 2011). qPCR analysis showed that at sphere stage *ccn1* transcripts in MZ*vgll4a* embryos were significantly reduced compared to those of wt, although this difference was no longer evident at 75% epiboly stages (Figure 4C). The mRNAs of the related *ccn2a* and *ccn2b* were instead undetectable at these stages.

These results suggest that maternal *vgll4a* function precedes yap activity. If this were the case, yap overexpression should rescue the phenotype of MZ*vgll4a* embryos. To test this possibility, we first compared the ability of human *YAP* mRNA and of its constitutively active YAP-5SA version (Zhao et al., 2007) to activate Yap/Taz-Tead responsive transgenic reporter line 4xGTIIC:eGFP (Miesfeld and Link, 2014). Both mRNAs efficiently activated eGFP expression in the reporter line (Fig. S6A-M) but *YAP-5SA* mRNA induced morphologically evident malformations in 24 hpf embryos (Fig. S6N-P), indicating that continuous and uncontrolled pathway activation has harmful effects. Based on these results, we next injected only *YAP* mRNA in MZ*vgll4a* embryos, which rescued their epibolic phenotype (Figures 4D, E) and partially rescued the A-P length of 3dpf larvae (Figure 4F).

Altogether these data indicate that maternal *vgll4a* is required to sustain yap1 signaling during zebrafish gastrulation.

### Maternal *vgll4a* is required for constriction of the actomyosin ring during epiboly

Loss or knockdown of *yap* in medaka, zebrafish and *Xenopus* embryo causes a delay in blastopore closure (Gee et al., 2011; Porazinski et al., 2015). In the *hir* medaka fish *yap* mutant, this delay is associated with a reduced actomyosin-mediated tissue tension, as also observed after the combined knockdown of *yap* and *taz* in zebrafish (Porazinski et al., 2015). Furthermore, in *hir* mutants the expression of the yap signaling effector, *arhgap18*, which controls tissue tension, is downregulated (Porazinski et al., 2015).

**FIGURE 5:**
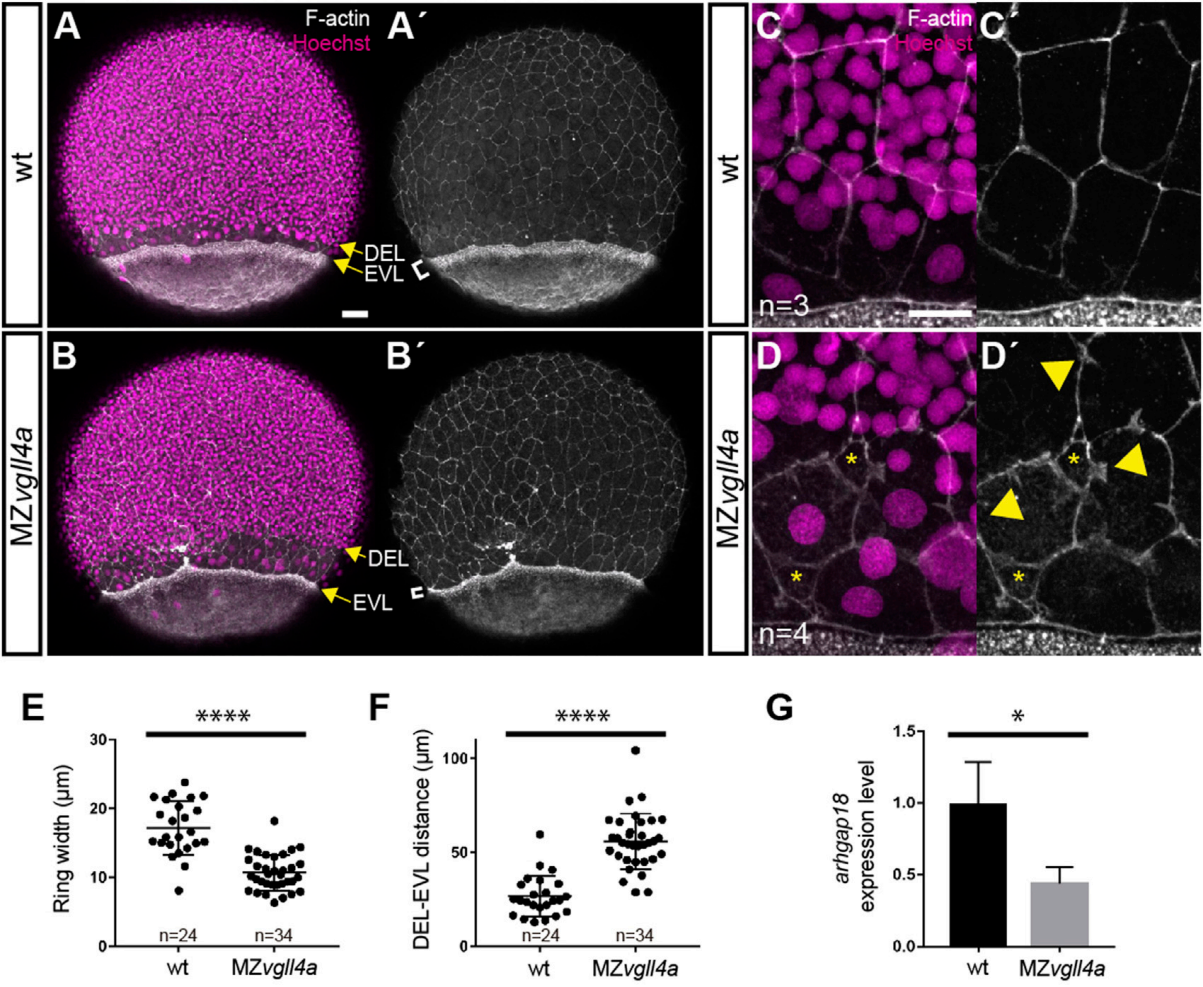
Maternal *vgll4a* is required for proper organization of the actomyosin ring. **(A-D′)** Confocal images of wt and MZ*vgll4a* embryos at 75% epiboly stage (lateral views) stained with phalloidin and Hoechst to visualize F-actin and nuclei, respectively. Note that the actomyosin ring in MZ*vgll4a* embryos is thinner (white brackets in A′, B′) and rather separated from the DEL margin (yellow arrows in A, B) as compared to wt embryos. Note also that F-actin distribution of EVL cells in MZ*vgll4a* embryos is ruffled and disorganized (yellow arrowheads in D′) in contrast to the well aligned distribution in wt embryos. Asterisk in D, D′ indicates loss of cell-cell contacts. **(E,F)** Quantification of the actomyosin ring width and DEL-EVL distance in wt (n = 24) and MZ*vgll4a* (n = 34) embryos. Mann-Whitney test, *p* < 0.0001). **(G)** The graphs show the expression level of *arhgap18* transcripts in MZ*vgll4a* and wt embryos at 75% epiboly stage. *t*-test, *p* = 0.041, as determined by qRT-PCR analysis. *, *p* < 0.05. ****, *p* < 0.0001. Scale Bars, a-b´ 50µm, c-d´, 20 µm.

We thus reasoned that if maternal *vgll4a* sustains Yap1 signaling, the MZ*vgll4a* phenotype should be also associated with alterations of the actomyosin ring and a reduction of the epibolic *arhgap18* expression. Indeed, in MZ*vgll4a* embryos, the actomyosin ring was thinner (Figures 5A, B′, E) and the DEL and EVL margins were further apart than those of wt embryos (Figures 5A, B′, F). The contour of EVL cells close to the EVL margin was also irregular with evaginations that resembled lamellipodia structures and loss of cellular contacts (Figures 5C, D′). Furthermore, and similarly to the *hir* mutants, the transcript levels of *arhgap18* were lower than those of wt (Figure 5G).

Taken together these observations support the idea that *vgll4a* function is a requisite for yap1-dependent actomyosin ring contractility and thus epiboly progression.

### Maternal *vgll4a* promotes E-cadherin/βcatenin distribution at the blastomere plasma membrane

Cells probe tension through plasma membrane proteins and then transmit the information to mechano-sensors such as Yap1 through cytoskeletal rearrangements (Dupont et al., 2011). Given that maternal *vgll4a* seemed to act upstream of yap1, we hypothesized that its activity could regulate blastomere adhesion and thus their tension probing capacity. Indeed in cancer cells, VGLL4 regulates the transcription of E-cadherin (Porazinski et al., 2015; Song et al., 2019) and the E-cadherin/α-Catenin/β-Catenin adhesion complex is an upstream regulator of Yap1 in different biological contexts (Kim et al., 2011; Schlegelmilch et al., 2011; Silvis et al., 2011). Furthermore, the epibolic phenotype of MZ*vgll4a* embryos resembled that of the pou5fl/Oct4 deficient MZ*spg* embryos (Lachnit et al., 2008; Song et al., 2013). In these mutants, deep cells move with a considerable delay in relation to the actin-depleted EVL margin, EVL cells form an abnormal number of lamellipodia (Lachnit et al., 2008, p. 2013; Song et al., 2013) and present a defective E-cadherin endosomal trafficking in blastomeres (Song et al., 2013). We thus compared the distribution of F-actin, β-catenin and E-Cadherin in wt and MZ*vgll4a* embryos, as read outs of blastomere cohesion (Yap et al., 2018).

**FIGURE 6:**
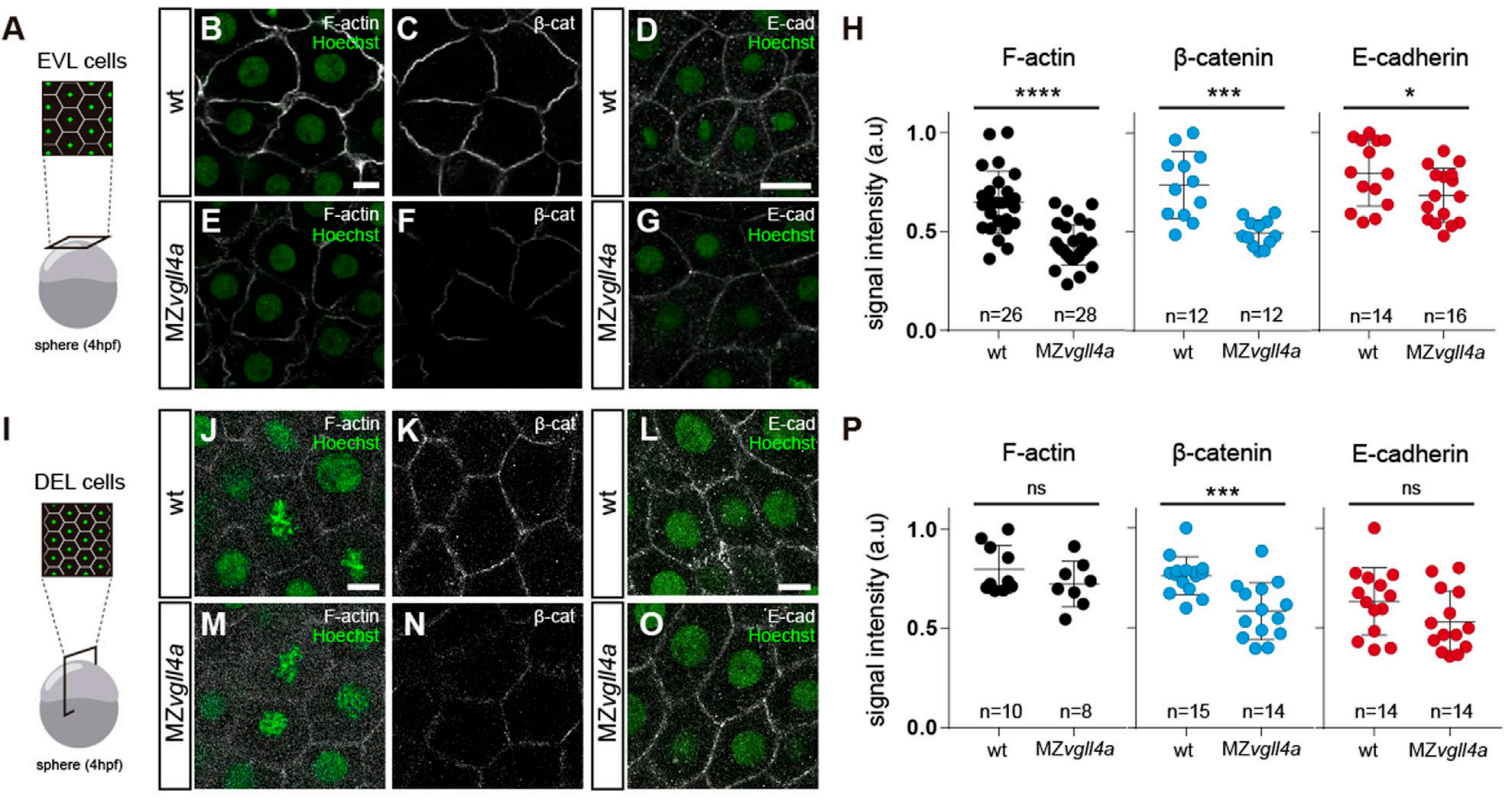
Maternal vgll4a is required for plasma membrane localization of the E-cadherin/β-catenin complex. **(A,I)** Schematic representation of the different imaging strategies. **(B-G; J-O)** Confocal images of F-actin **(B, E, J, M)**, β-catenin **(C, F, K, N)** and E-cadherin **(D, G, L, O)** distribution in EVL **(B–G)** and DEL **(J–O)** cells in wt and MZ*vgll4a* embryos at sphere stage. Embryos were counterstained with Hoechst (nuclei, green). **(H,P)** The graphs depict the fluorescent signal intensity (in arbitrary units, a. u) for F-actin, β-catenin and E-cadherin in EVL **(H)** or DEL **(P)** cells of wt and MZ*vgll4a* embryos. Mann Whitney test. **(H)** β-catenin, *p* = 0.0001, E-cadherin; *p* = 0.0425. **(P)** F-actin, *p* = 0.3154; β-catenin, *p* = 0.0006 and E-cadherin, *p* = 0.1599. Ns, not significant, *p < 0.05; ***p < 0.001; ****p < 0.0001. Scale bar, 10 µm.

F-actin, β-catenin and, to a lesser extent, E-cadherin signal intensity of MZvgll4a EVL cells was significantly decreased at sphere stage (Figures 6A–H). These changes were less evident in DEL cells, where only β-catenin was significantly reduced at sphere (Supplementary Figures S6I-P) and shield (Supplementary Figure S7) stages. Nevertheless, the overall E-cadherin mRNA expression levels (cdh1) in wt and MZvgll4a embryos were similar at both sphere and shield stage (Supplementary Figures S8A, C). β-catenin acts as an effector of Wnt signaling and thus its decreased levels at the plasma membrane could be a consequence of an over-activation of the pathway. To test this possibility, we determined the expression levels of different Wnt targets and read-outs. No difference was observed in the levels of axin1, axin2 and lef1 expression between MZvgll4a and wt embryos at both sphere and shied stage (Supplementary Figure S8B, D).

All in all, these data indicate that maternal vgll4a promotes E-cadherin/β-catenin localization at the blastomeres’ plasma membrane and hence their actin cortex distribution.

## Discussion

The genome of a fertilized egg is transcriptionally inactive. Thus, maternal RNAs and proteins present in the eggs are responsible for coordinating the first morphogenetic events that takes place during gastrulation, initially single-handed and then in cooperation with the progressively available zygotic gene products (Solnica-Krezel, 2020). In the zebrafish egg, these maternally derived molecules represent a large proportion of all possible gene products (Harvey et al., 2013). However, how many of them are critical for epiboly initiation and progression is still poorly defined. Genome wide (Kane et al., 1996) and specific maternal-effect mutational screens in zebrafish have identified only a limited number of mutants with an epibolic phenotype (Dosch et al., 2004; Wagner et al., 2004) and their subsequent characterization supports an important contribution of adhesion molecules and cytoskeletal components (Bruce, 2016). Other studies have thereafter identified a few maternally inherited transcriptional regulators, including eomes/tbr2, foxh1, pou5fl/oct4, nanog and yap1 (Bruce et al., 2005; Reim and Brand, 2006; Pei et al., 2007; Lachnit et al., 2008; Song et al., 2013; Porazinski et al., 2015; Gagnon et al., 2018; Veil et al., 2018). In this still fragmented scenario, our study adds a new component to the genetic network coordinating zebrafish epiboly, showing an important role for maternally inherited *vgll4a*, and, to some extent, for its paralog *vgll4b*. Notably, it also shows that vgll4a is required to support yap1 signalling and the expression of E-cadherin/β-catenin adhesion complex (directly or indirectly), thereby coordinating blastomere adhesion/cohesion. This observation aligns with recent studies demonstrating the versatility of Vgll4 in various biological contexts, extending beyond its established role as a Yap1 inhibitor (Fillatre et al., 2019; Xue et al., 2019; Quan et al., 2023; Wang et al., 2023).

Zebrafish epiboly can be divided in two phases: initiation or doming and progression. Our study underscore *vgll4a* requirement at doming with morphological consequences that become visible as epiboly progresses and later as the embryonic A-P axis extends. Our findings are substantiated by the distribution of *tbxta*, a marker for margin cells, at 4.5 and 8hpf. Additionally, disrupting myosin activity precisely at the 4hpf stage replicates the MZ*vgll4a* mutant phenotype, both during epiboly and in larval stages. This observation establishes a functional link between these two events. A recent study strongly supports this possibility demonstrating reduced *Xenopus* A-P axis extension when tissue scale force production is wakened during gastrulation (Huebner et al., 2022). Furthermore, a short axis phenotype was also observed after interference with myosin activity in MZ*alkbh4* and M*alkbh4* mutants (Sun et al., 2017), in which defective convergent extension movements were also reported. Thus, it is plausible that similar gastrulation defects may contribute to the phenotype observed in MZ*vgll4a* embryos, although we have not investigated this possibility.

The large majority of factors implicated in doming are maternally expressed, as both *vgll4a* and *vgll4b* are (Xue et al., 2018). The time-lag between the postulated molecular activity of maternal vgll4a at doming and the phenotypic consequences about 4 hours later is also not surprising, as a similar delay has been reported for other factors contributing to epiboly initiation (Lachnit et al., 2008; Sun et al., 2017). A recent study has shown that at sphere stage the central blastula becomes “fluid” as a consequence of loss of cell-cell adhesion and increased cell division (Petridou et al., 2019). The blastoderm margins instead maintain cell-cell adhesion and thus tension thanks to the activity of wnt11 non-canonical signalling (Petridou et al., 2019), known to control E-cadherin availability at the plasma membrane (Ulrich et al., 2005). If this differential fluid vs. tense state is perturbed, the ability of the blastula to react to mechanical forces is altered and epiboly becomes defective (Petridou et al., 2019). In our experiments we have not detected changes in wnt signalling using the global expression of three different downstream targets. Furthermore, vgll4a role in epiboly seems to be strictly maternal (Solnica-Krezel, 2020) and thus could act upstream of wnt11 signalling. Vgll4a-mediated control of wnt signalling will impinge on E-cadherin/β-catenin availability at the plasma membrane and thereby on differential blastoderm viscosity that favours epiboly progression. In support of this possibility, *vgll4* paralogs appear to control the expression of non-canonical wnt signaling components, including *wnt11f2*, *fzd8a* and *fzd10* (Fillatre et al., 2019). Nonetheless, we cannot exclude that very local changes in wnt pathway activation may occur, thereby explaining the MZ*vgll4a* mutants phenotype as proposed by Petridou et al. (Petridou et al., 2019). Alternatively, vgll4a could interfere with non-canonical wnt signaling indirectly through the regulation of wnt/β-catenin canonical pathway, as the two branches of the pathway can have antagonistic effects in morphogenesis (Cavodeassi et al., 2005). Indeed, VGLL4 seems to interfere with the formation of a TEAD4-TCF4 complex, which promotes cell proliferation in colorectal cancer (Jiao et al., 2017). Furthermore, VGLL4 overexpression suppresses nuclear β-catenin levels and inhibits migration and invasion of gastric cancer cells, while its inactivation has opposite effects (Li et al., 2015). As an additional possibility, vgll4a could directly regulate E-cadherin expression as observed in both gastric and breast cancers (Li et al., 2015; Song et al., 2019), thereby influencing the membrane levels of E-cadherin/β-catenin complexes. According to our data the latter possibilities are however less likely. We observed a small, although significant, decrease of E-cadherin membrane localization only in EVL cells of MZ*vgll4a* embryos at sphere stage, and no significant changes in the mRNA levels of *cdh1* or in those of three read-out of wnt/β-catenin signalling, but our analysis, performed with entire embryos, precludes the identification of changes present only in a small cell subset. Furthermore, we cannot rule out that the MZ*vgll4a* phenotype might be associated with abnormal E-cadherin stability or trafficking, as reported for the zebrafish *wnt11f2* and *oct4/*MZ*spg* mutants (Song et al., 2013; Petridou et al., 2019). This destabilization, in turn, could impinge on the actin cortex, which seems incomplete in most EVL cells (Figure 6), and thereby on the integrity of tight junctions. Although we have not addressed this point, the morphology of the EVL layer in MZ*vgll4a* mutants resembles that of embryos treated with the dynamin inhibitor Dynasore (Lepage et al., 2014) or that reported for E-Cadherin mutants (Lachnit et al., 2008).

We focused on *vgll4a* and report that the related *vgll4l* has no role in epiboly, consistent with its lack of early expression (Xue et al., 2018) and its suggested functional diversification (Fillatre et al., 2019). On the contrary, we show both *vgll4a* and *vgll4b* seem to be important for oocyte fecundity and that MZ*vgll4b* and MZ*vgll4a* mutants present a very comparable A-P axis phenotype. However, in contrast to what observed for *vgll4a*, maternal *vgll4b* activity can be compensated by its zygotic counterpart. Furthermore, MZ*vgll4a;*MZ*vgll4b* double mutants are phenotypically very comparable to the two single mutants, and the additional inactivation MZ*vgll4l* does not significantly worsen the short axis phenotype, compatible with the absence of maternal *vgll4l* expression. This suggests that the different paralogs do not compensate each other function and that *vgll4a* and *vgll4b* perhaps act in a different spatio-temporal window that converges in the same shorter axis phenotype. At later stages of development, *vgll4b* has been implicated in erythropoiesis, terminal differentiation (Wang et al., 2020), heart valvulogenesis (Xue et al., 2019) and establishment of the left-right asymmetry in coordination with *vgll4l* (Fillatre et al., 2019)*.* Our *vgll4a* mutants do not show similar traits, at least in a gross morphological analysis, further supporting a possible diversification of the paralogs’ activity.

Vgll4 has been initially described as co-transcriptional repressor that interacts with Tead transcription factors, thereby acting as an as antagonist of Yap signalling (Guo et al., 2013; Zhang et al., 2014). This function has been thereafter validated in multiple contexts especially in tumour development (Yamaguchi, 2020). Our study not only shows that maternal vgll4a is required for epiboly but also that does so supporting rather than antagonising Yap1 signalling. The generation of a transgenic Yap/Taz-Tead reporter zebrafish line faithfully highlights pathway activation from 22 hpf (Miesfeld and Link, 2014). Using this same line we have been unable to detect reporter expression at epiboly stages. However, we show that overexpression of YAP is sufficient to rescue the phenotype of MZ*vgll4a* mutants, suggesting that, likely, maternal vgll4a acts upstream of Yap1, given that *ccn1* expression is decreased in MZ*vgll4a* mutants. Furthermore, addition of the Yap inhibitor verteporfin to wt embryos replicates the MZ*vgll4a* phenotype but has no effect on the MZ*vgll4a* mutants themselves.

Our analysis has been performed at the whole embryo scale, taking epiboly progression, actomyosin organization and cell cohesion as read-out of *vgll4a* function in morphogenetic rearrangement. These rearrangements highly depend on mechanical and biochemical events at the cellular and subcellular scale (Petridou et al., 2017; Stooke-Vaughan and Campàs, 2018). Therefore, in this scenario, we cannot exclude that the delay in epiboly progression observed in MZ*vgll4a* embryos represents the macroscopic result of a series of mechano-chemical feedback loops at the cellular scale (Hannezo and Heisenberg, 2019), involving both vgll4a and yap1 in the same or even different cell populations.

**FIGURE 7:**
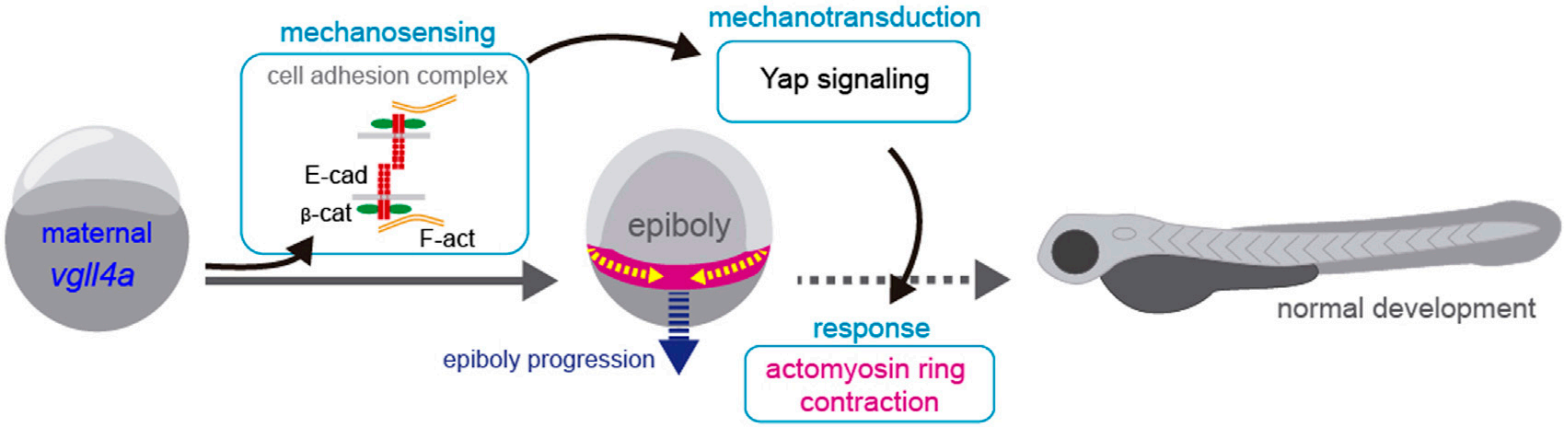
Proposed model for maternal vgll4a contribution to zebrafish epiboly progression. Maternal *vgll4a* promotes plasma membrane localization of the E-cadherin/β-catenin complex in the amount required for an adequate cohesion among blastomeres. This cohesion threshold allows tissue mechano-sensing and thus yap1-dependent mechano-transduction. Signal transduction impacts on the organization and function of the actomyosin ring, thereby promoting timely epiboly progression. E-cad, E-cadherin; β-cat, β-catenin; F-act, F-actin.

Nevertheless, and independently of the relative position of *vgll4a* and *yap1* in epiboly regulation, our data suggests that vgll4a may be required for a proper balance between tissue tension/cohesion and contractility, two factors that contribute to mechanical stress (Wozniak and Chen, 2009). Indeed, decreasing myosin contractility at doming mimics the absence of maternal *vgll4a* function. We thus hypothesis that in the absence of *vgll4a*, cellular cohesion mediated by adhesion complexes is poor, impairing embryonic mechano-sensation (Figure 7). This impairment would decrease yap-dependent mechano-transduction, which, in turn, could affect the contraction of the actomyosin network, delaying epiboly progression (Figure 7). In support of this model, the yap mechano-regulatory program seems to be essential for sustaining intracellular tension during gastrulation, that, in turn, controls the assembly of the embryonic A-P axis (Sousa-Ortega et al., 2023). Our simplified model does not take into account other and yet undetermined factors that contribute to this process. For example, we were unable to rescue the speed of epibolic movements in MZ*vgll4a* embryos with calyculin, a drug that increases myosin 2 contractility, either because there are other compensatory mechanisms or because we have not chosen the appropriate time window.

Independently from these considerations and in a broader context, our data suggest that upregulation of *vgll4* expression may serve to enhance the mechano-sensing properties of some tissues, perhaps restoring an unbalanced back-and-forth dialogue between biochemical and mechanical cues, which has been described in pathological conditions, including different type of primary and metastatic cancers (Deng and Fang, 2018).

## Materials and methods

*Maintenance of fish lines*. Adult AB/TUE wild type, mutant (*vgll4a, vgll4b, vgll4l*) and 4xGTIIC:eGFP reporter line (Miesfeld and Link, 2014) zebrafish were maintained at 28°C on 14/10 h light/dark cycle. Embryos were raised at 28°C and staged according to the hours post fertilization (hpf) and their morphology. Embryos were growth in E3 medium (NaCl, 5 mM; KCl, 0.17 mM; CaCl_2_, 0.33 mM; MgSO_4_, 0.33 mM; 5.10% Methylene Blue). The ethical committee for Animal Experimentation of the Consejo Superior de Investigaciones Científicas (CSIC) and of the Comunidad Autónoma de Madrid approved the procedures used in the study.

*Zebrafish mutants generation*. Zebrafish mutant lines were generated using CRISPR/Cas9 technology. The gRNA were designed using the CHOP-CHOP tool (Labun et al., 2019) looking for potential disruption of enzyme restriction sites. gRNAs were synthesized using PCR templates as described in (Varshney et al., 2016). Cas9 protein (300 ng/μL; EnGen^®^ Spy Cas9 NLS, New England Biolabs) and gRNAs (100 ng/μL; Table S1) were co-injected into one-cell stage zebrafish embryos. F0 embryos were raised and outcrossed with AB/TUE wt. PCR amplification on genomic DNA isolated from tail clips of F1 zebrafish embryos was performed to identify disruption of specific restriction sites (Table S2). The DNA of potential mutants was thereafter sequenced and the selected embryos were raised to adulthood. We selected mutants in which the reading frame was disrupted and truncated as follow: *vgll4a*-S85Mfs15, *vgll4b*-P156Rfs5 and *vgll4l* P111Hfs61 (see Supplementary Figure S1 for more details).

*Embryo injections*. Zebrafish embryos were injected at the 1 cell stage with 1 nL of the selected solutions using a IM-300/Narishige microinjector. Recombinant humanVGLL4 protein (0.19 ug/μL; Abnova, Ref: H00009686-P01) and *vgll4a*-HA mRNA (100 ng/μL) were used in rescue experiments. Phosphate buffer saline (PBS) containing 0.1% BSA or mutated *vgll4a-*HA mRNA (cloned from the *vgll4a*-S85Mfs15 line) were injected as controls. Myc-YAP, Myc-YAP-5SA and eGFP mRNAs were injected at 25 ng/ul.

*Tissue processing and immunohistochemistry*. Embryos were fixed by immersion in 4% paraformaldehyde in 0.1 M phosphate buffer pH 7.2 (wt/vol) overnight at 4 °C. Embryos were then washed in PBS with 0.5% Triton-X-100, incubated in a 15% sucrose-PBS solution (wt/vol), embedded and frozen in a 7.5% gelatin in 15% sucrose solution (wt/vol). Cryostat sections or whole embryos were stained using standard protocols and antibodies against the following antigens: HA (1:250, Sigma, H-6908), β-catenin (1:300, BD Bioscience, 610,154), E-cadherin (1:300, BD Bioscience, 610,181) and Yap (1:200, Cell Signaling Technology, #4912). Incubation with Phalloidin-TRITC (1:200, Sigma, P1951) was used to detect actin. Incubation with appropriate secondary antibodies was performed following standard procedures.

*In situ hybridization* (*ISH*)*.* A specific *tbtxa* probe was generated by RT-PCR from cDNA of 6hpf embryos using the following primers (5′-GAT​CGG​AAA​TAT​GTC​TGC-3′ and 5′-GTTGTCAGTGCT GTGGTC-3′) with the Expand™ High Fidelity PCR System (Roche) and cloned with the StrataClone PCR Cloning Kit (Agilent). *myod1* RNA probe was synthesized from cDNA encoding *myod1* (Weinberg et al., 1996)*.* Digoxigenin-UTP-labelled antisense riboprobes were synthesized *in vitro* using DIG RNA labelling Mix kit (Roche). Zebrafish embryos at different developmental stages were hybridized by standard procedures and visualized with NBT/BCIP (dark blue).

*Construct generations*. *vgll4a* was amplified from cDNA of wt or *MZvgll4a* (as control) embryos using the following primers (Fw: 5′-GGA​ATC​AAC​AGT​TAG​CGT​GCT-3’; Rv: 5′-aaC​TCG​AGT​CAA​GCG​TAA​TCT​GGA​ACA​TCG​TAT​GGG​TAA​GAC​TGA​CCA​ACA​TGA​TTG-3′). The amplicon, includes the hemagglutinin (HA) epitope included in the reverse prime, was cloned by TA-cloning in the pSC-A vector using the StrataClone PCR Cloning Kit (Agilent). The *vgll4a-HA* fragment was thereafter liberated from the pCS-A construct using EcoRI/XhoI digestion and cloned in the pCS2 vector. The pCS2 construct, linearized with NotI, was used to synthesize mRNA from the wt or mutated *vgll4a* version, tagged with HA, using the mMESSAGE mMACHINE SP6 Transcription kit (Invitrogene) following manufacturer’s instructions. After transcription, mRNAs were purified using the NucleoSpin^®^ RNA Clean-up kit (Machery Nagel). pQCXIH-Myc-YAP (Addgene plasmid # 33091) and pQCXIH-Myc-YAP-5SA (Addgene plasmid # 33093) (Zhao et al., 2007) were used to clone the Myc-tagged forms of human YAP in the pCS2 vector, after NotI (Takara) and EcoRI restriction digestion. The open pCS2 vector and the fragments were ligated using T4 DNA Ligase (NEB) and the resulting constructs used to synthesize the corresponding mRNA.

*Quantitative RT-PCR analysis*. Total RNA was isolated from wt and mutant embryos (n = 30, for each genotype) using TRIzol (Sigma) according to the manufacturer´s instruction. Each experiment was performed with biological triplicates. 5 μg of total RNA was used to synthesize the first-strand cDNA using the First-Strand cDNA synthesis kit (GE Healthcare) with a pd(N)_6_ primer. Each quantitative RT-qPCR reaction was performed using the GoTaq qPCR Master Mix kit (Promega). For a 10 µL reaction, 4 µL of cDNA (2.5 ng/μL) was mixed with 1 µL of primers (2.5 µM; Table S3) and 4 µL master mix. Reaction was incubated at 95°C for 10 min, then at 95°C for 15 s and 40 cycles and at 60°C for 60 s. The levels of the *eef1a1l1* mRNA were used as housekeeping reference (Xu et al., 2016).

*Pharmacological treatment*. Embryos were incubated for different time-windows at 28°C in E3 medium containing appropriate volumes of DMSO alone or DMSO containing either Blebbistatin (203,390, Calbiochem) Calyculin A (BML-EI192, Enzo). or Verteporfin (5,305, Tocris). Treatment with 50 µM Blebbistatin was carried out for 30 min using three different time-windows (3, 4 and 5 hpf). Verteporfin (5 µM) was added to embryos at the 1 cell stage up to 75% epiboly (8 hpf). Calyculin A was used at four different concentrations (0.2 µM, 0.5 µM, 0.7µM and 1 µM) and added at 4hpf for 30 min.

*Imagin*g. Sections were analysed with DM or confocal microscope. Zebrafish embryos at different stages were observed and photographed using a stereomicroscope and DFC500, DFC350 FX cameras (Leica Microsystems). For sections or whole embryo staining, LSM710 confocal laser scanning microscope coupled to an AxioObserver inverted microscope (Zeiss) were used to obtain digital images, which were then processed and analysed with ImageJ (Fiji) software. Images shown in Figure 1, Figure 4, Figure 5, Fig. S1, S4 and S6 were assembled using the Photoshop CS5 software.

*Quantification and statistical analysis*. All quantifications were performed using the ImageJ (Fiji) software. 72hpf embryonic larvae were treated with tricaine and photographed in lateral views at ×13 magnification to determine their body length. For epiboly progression quantifications, embryos hybridized with a *tbxta* probe were photographed and the total length of the egg and the distance between the margin cells and the vegetal pole were measured. Blastopore closure quantification was performed as previously reported (Köppen et al., 2006). The area of individual and centrally positioned blastoderm cells from wt and mutant embryos was determined by tracing their perimeter on confocal images of whole embryos stained with β-catenin antibody. Only one image per embryo was used. The position and width of the actomyosin ring was determined on z-stack files obtained from the confocal images of whole embryos. The z-stack files were used to generate maximum intensity projections. The mid-region of the actomyosin ring and DEL/EVL margins were positioned in the centre of the image to avoid possible visual distortions due to the embryo curvature and a rectangular region of interest (ROI) was selected covering the central area of the embryo. Ten different measurements of the width of the actomyosin ring as well as ten different measurements of the distance between DEL-EVL were obtained along the ROI using the “straight” selection tool. The ten measurement *per* embryo were averaged to obtain a more accurate value of the ring width and also the DEL-EVL distance and their means were used in the comparative analysis. Immunofluorescence intensity quantifications were performed using mid-plane of z-stack files obtained from the confocal imaging of β-catenin, F-actin and E-cadherin staining on whole embryos (EVL cells) or cryostat sections of different embryos (DEL cells). A rectangular ROI was used to measure the mean grey value of the selected image as indicator of signal intensity. GraphPad Prism 7 statistic software was used to analyze the data using *t*-test for two groups with parametric distribution and with Mann-Whitney test for two groups with no parametric distribution. One-way ANOVA test was used for more than two groups with parametric distribution with Tukey’s multiple comparisons test to analyze differences between groups. Kruskal–Wallis test for no parametric distribution with Dunn’s multiple comparisons test to analyze differences between groups. Fisher exact test was used in the analysis of Figure 1G. Statistical difference between pools of treated and control embryos was determined with the Pearson´s X2 test. Error bars indicate s. e.m. in all graphs.

## Data Availability

The original contributions presented in the study are included in the article/Supplementary Material, further inquiries can be directed to the corresponding author.
