# Supplementary material for: Maternal vgll4a regulates zebrafish epiboly through Yap1 activity
Source: Front Cell Dev Biol. 2024 Feb 20;12:1362695. doi: 10.3389/fcell.2024.1362695 (PMC10912589; doi:10.3389/fcell.2024.1362695)
Supplement: Supplementary file 1 [file DataSheet1.PDF]

Maternal *vgll4a* regulates zebrafish epiboly through Yap1 activity.

Carlos Camacho-Macorra[1,2,&], Noemí Tabanera[1,2], Elena Sánchez-Bustamante[1,2], Paola Bovolenta[1,2,* #] and Marcos J Cardozo[1,2, *]

[1]*Centro de Biología Molecular Severo Ochoa, Consejo Superior de Investigaciones Científicas-Universidad Autónoma de Madrid, Madrid 28049, Spain*

[2]*Centro de Investigación Biomédica en Red de Enfermedades Raras (CIBERER), Madrid 28049, Spain*

*- Last co-authors

Corresponding author: pbovolenta@cbm.csic.es

[&]**Current address***: Dept Genetics, University of Cambridge, Cambridge CB2 3EH, UK*

Running title: *vgll4a* function in epiboly progression

Keywords: Yap signaling, Vgll4, *vgll4a*, actomyosin, E-cadherin complex, epiboly

Supplementary Figures S1-S8

Supplementary Tables S1-S3

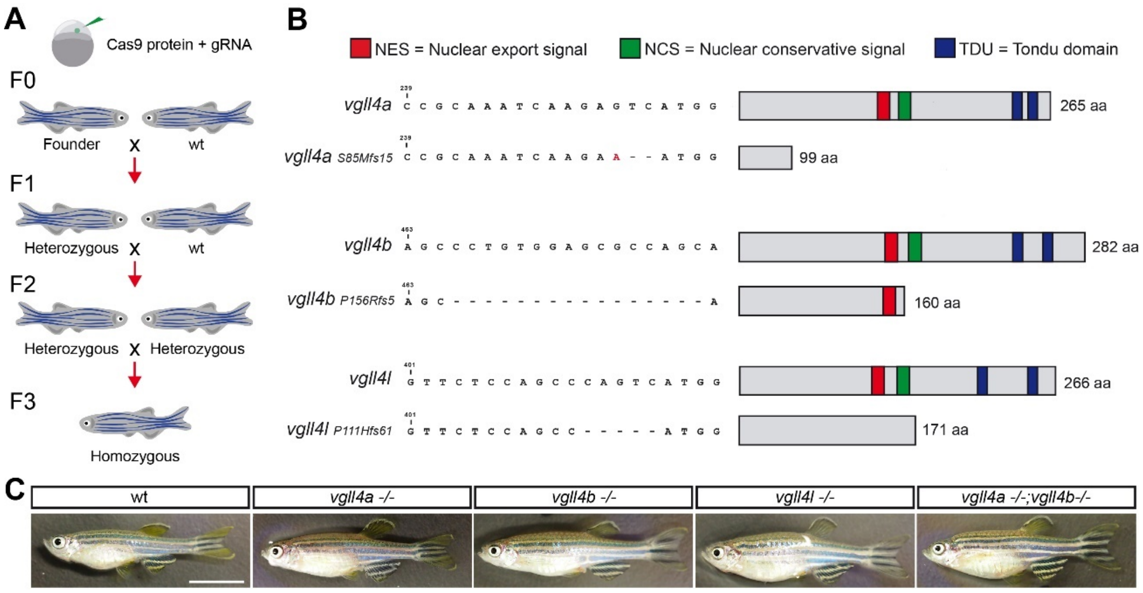

Figure S1. Generation and characterization of *vgll4a*, *vgll4b* and *vgll4l* zebrafish mutant. A) Schematic representation of the strategy used to generate *vgll4a*, *vgll4b* and *vgll4l* zebrafish mutants from the injection of specific gRNAs and Cas9 protein into one cell stage embryos to the generation of F3 adult homozygous mutant fish. B) Schematic representation of the zebrafish Vgll4 proteins with its characteristic domains and the expected truncated versions in the generated *vgll4a* S85Mfs15, *vgll4b* P156Rfs5 and *vgll4l* P111Hfs61 zebrafish mutants. C) Bright field images of wt, *vgll4a*, *vgll4b*, *vgll4l* and *vgll4a;vgll4b* adult homozygous mutants at three months of age.

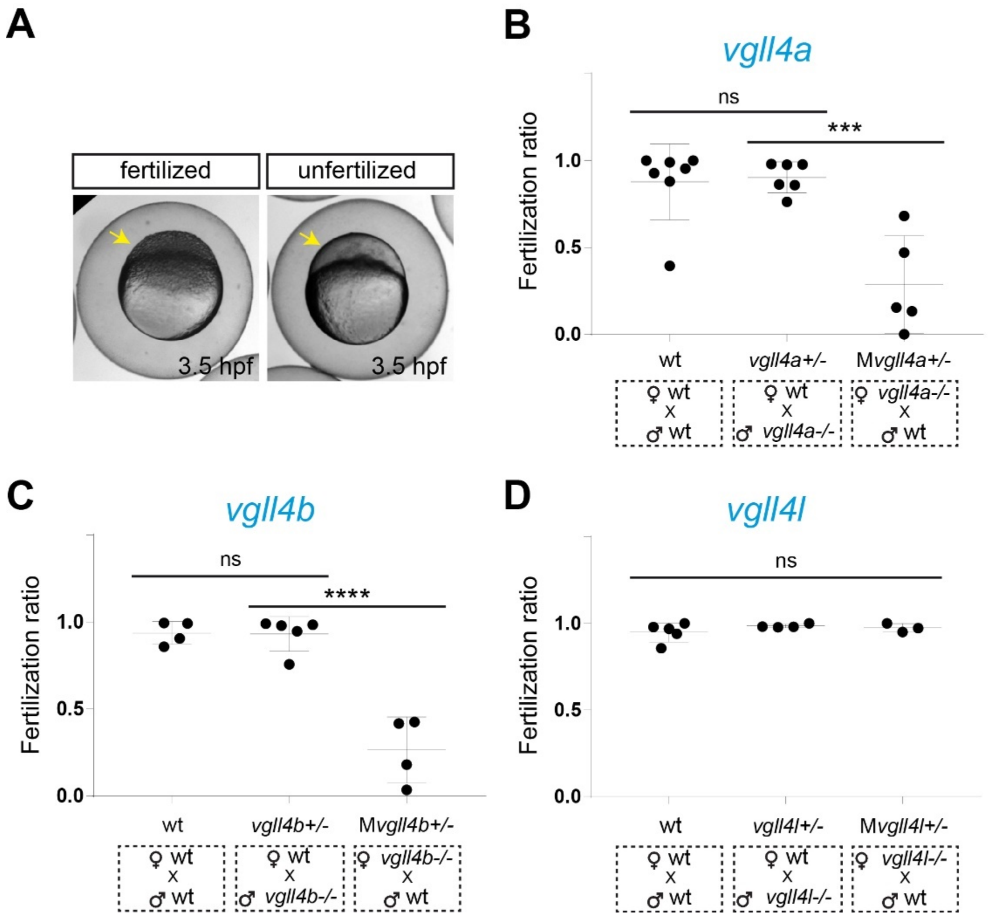

Figure S2. Maternal vgll4a and vgll4b, but not vgll4l, support egg fertility. A) Bright field images of a fertilized and unfertilized egg at 3.5hpf (the yellow arrows point to the animal pole). B-D) The graphs show the ratio of fertilization in eggs from wt, *vgll4a* (B) *vgll4b* (C) and *vgll4l* (D) heterozygous genotypes with or without vgll4 maternal contribution. The ratio represents the number of fertilized eggs over the total number of eggs in a clutch. Each point represents a different clutch, which was composed of 20 eggs minimum. The dashed boxes below the graphs indicate the sex and genotype of the fishes mated to obtain the desired genotypes. Note that M*vgll4a*+/- and M*vgll4b*+/- eggs are less fertile than those of wt or with *vgll4a* or *vgll4b* maternal contribution (One-Way ANOVA. $p= 0.0002$ for B and $p< 0.0001$ for C). In contrast, M*vgll4l*+/- eggs lacking maternal *vgll4l* present a similar fertilization ratio than those of wt or with *vgll4l* maternal contribution. (one-Way ANOVA. ns, not significant).

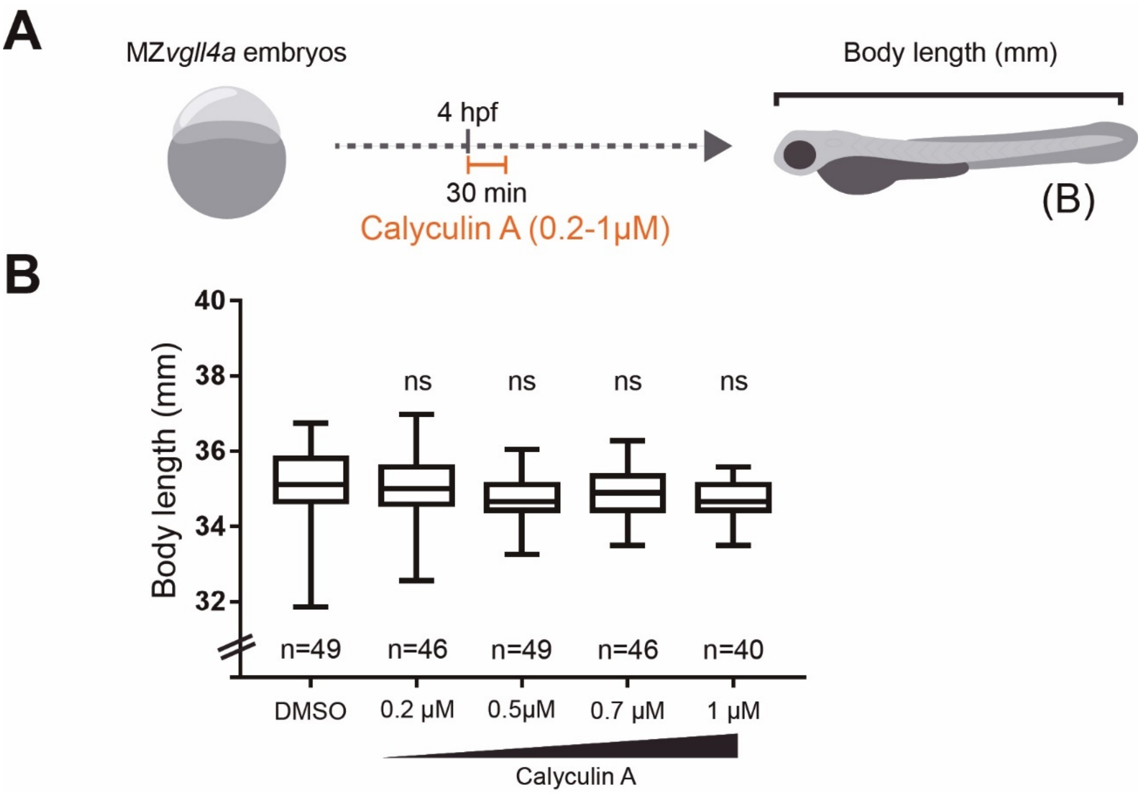

Figure S3. Myosin activation does not rescue the body size of MZ*vgll4a* larvae. A) Schematic representation of the experimental design. B) Box plots of the larva body length from 3dpf MZ*vgll4a* embryos grown in the presence of DMSO or Calyculin A at different concentrations. Data were analyzed with Kruskal-Wallis test. 0.2uM, $p>0.9999$; 0.5uM, $p=0.1916$; 0.7uM, p>0,9999; 1uM, $p=0.1915$. ns, not significant.

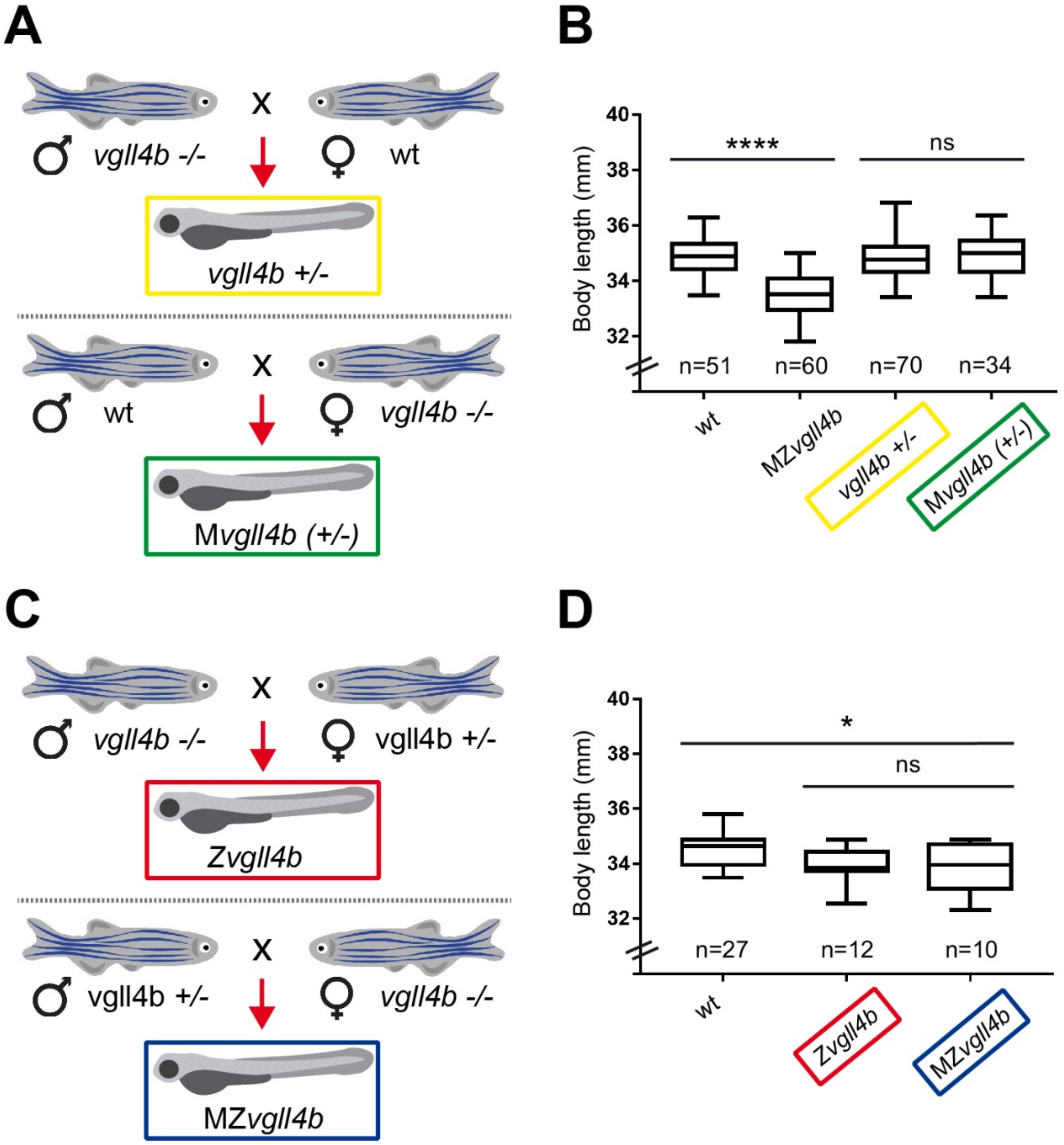

Figure S4. Maternal *vgll4b* is dispensable for embryonic growth. A, C) Schematic representation of the mating strategy used to obtain embryos of the desired genotypes with or without *vgll4b* maternal contribution. B, D) Box plots of the body length from 3dpf larvae of the indicated genotypes. Note the decreased body length of embryos in the absence of zygotic vgll4b as compared to wt or heterozygous embryos. The number of analyzed embryos is indicated below each plot. Data in B and D were analyzed with One-Way ANOVA test; * $p<0,05$; **** $p<0.0001$.; ns, not significant (in B wt vs. *vgll4b*+/-, $p=0.4006$; wt vs. M*vgll4b*+/-, $p=0.9874$; in D wt vs. Z*vgll4b*, $p= 0.0297$; wt vs. MZ*vgll4b*, $p= 0.0315$; Z*vgll4b* vs. MZ*vgll4b*, $p= 0.9917$).

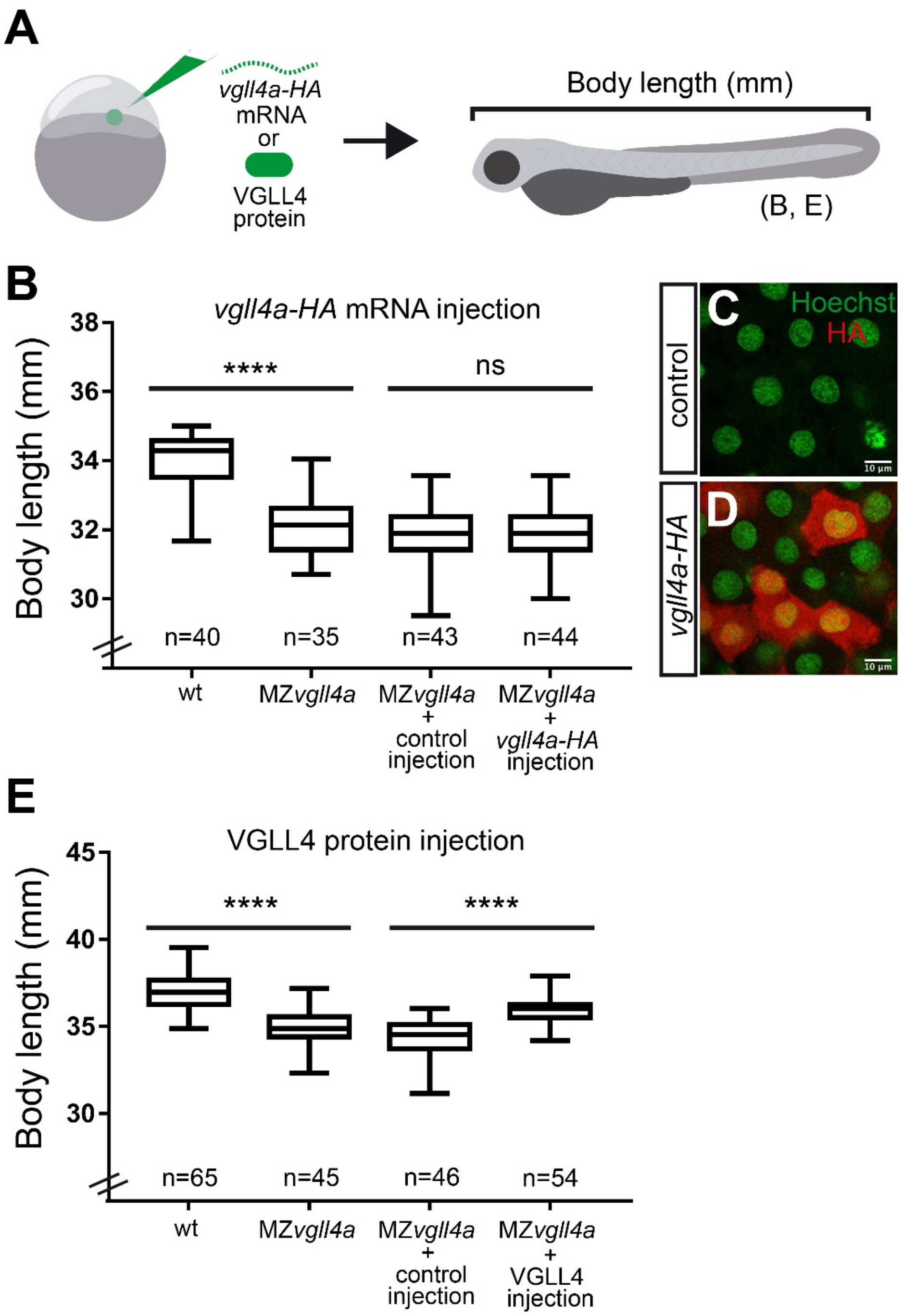

Figure S5. VGLL4 rescues MZvgll4a embryonic developmental delay. A) Schematic representation of the experimental design used to demonstrate the direct relationship between vgll4 and embryonic growth. B, E) The graphs show the body length of wt and MZ*vgll4a* embryos injected with control and *vgll4a*-HA mRNA (B) or VGLL4 protein (E). Note that only protein injection can rescue the maternal effect of vgll4a. One-Way ANOVA. ns, not significant. **** p<0.0001. Wt vs. MZ*vgll4a* VGLL4 injected embryos, p< 0.0001. MZ*vgll4a* vs. MZ*vgll4a* VGLL4 injected embryos, p< 0.0001. C, D) Confocal images of MZ*vgll4a* embryos with (D) or without (C) *vgll4a*-HA mRNA and immunostained with anti-HA antibodies. Note the mosaic expression of *vgll4a*-HA.

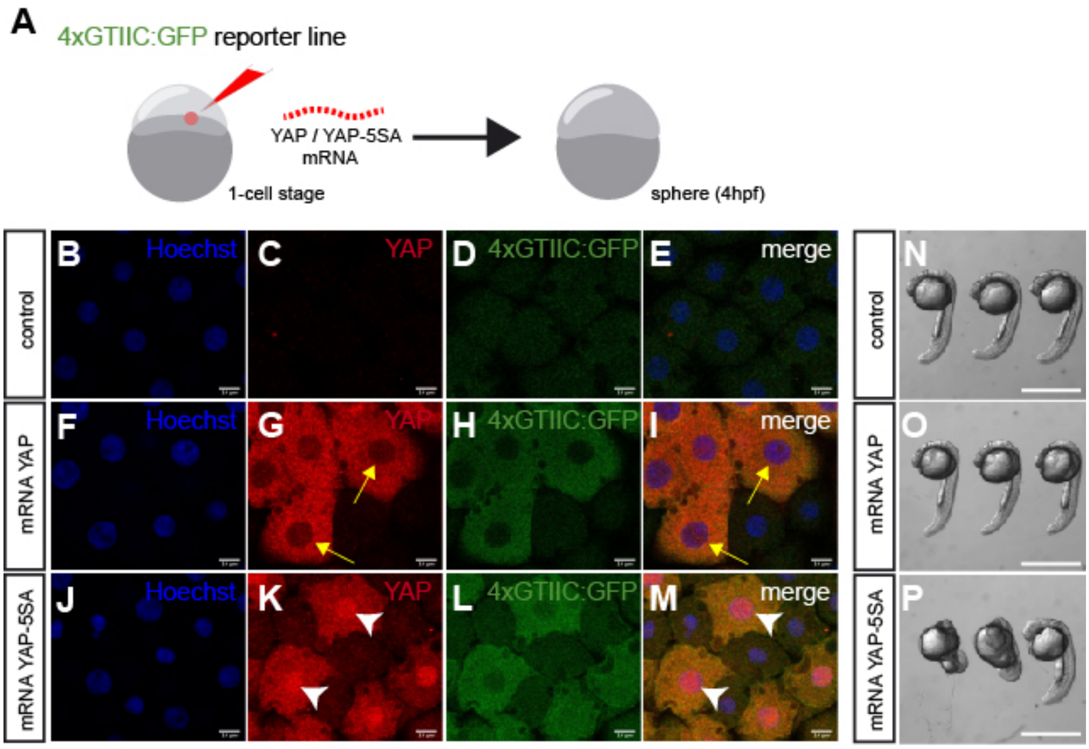

Figure S6. YAP overexpression efficiently activates Yap reporter expression in transgenic embryos. A) Schematic representation of the experimental design used to test the efficiency of YAP overexpression. B-M) Confocal images of transgenic 4xGTIIC:GFP embryos at sphere stage (dorsal views) stained with Hoechst to visualize nuclei (B, F, J) and with anti-Yap antibody (C, G, K). In contrast to the absence of reporter expression observed in control embryos (B-E), the injection of 25pg of YAP mRNA (F-I) or of its constitutively activated YAP-5SA version (J-M) efficiently activated the 4xGTIIC:GFP reporter line (H, L). Note that YAP localizes mostly in the cytoplasm (arrows in G, I,) whereas YAP-5SA in the nuclei (arrowheads in K, M). N-P) In contrast to YAP-5SA, YAP overexpression does not alter the gross morphology of the embryos at 24hpf. Scale bar (N-P): 1mm.

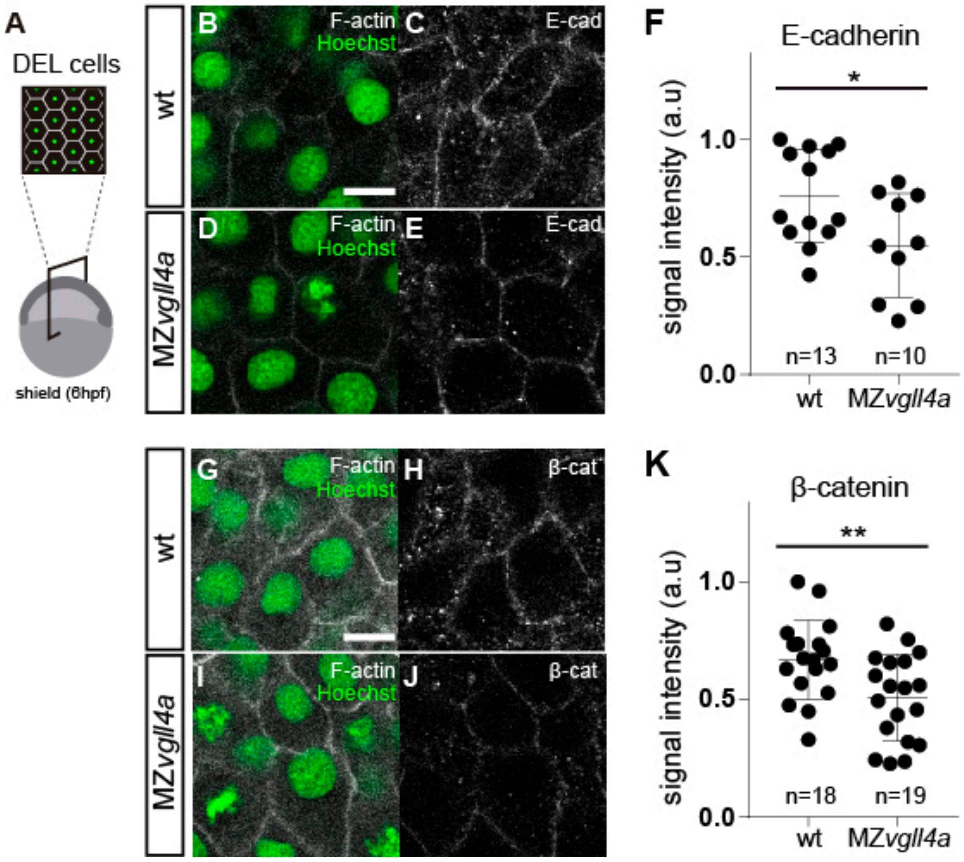

Figure S7. ***Vgll4a*** **is required for plasma-membrane localization of β-catenin at shield stage**. A) Schematic representation of the imaging strategy. B-E; G-J) Confocal images of E-cadherin (C, E) and β-catenin (H, J) distribution in DEL cells of wt and MZ*vgll4a* embryos at shield stage. Embryos were co-stained with phalloidin (F-actin) and Hoechst (nuclei, green) (B, D, G, I). F, K) The graphs depict the fluorescent signal intensity (in arbitrary units, a.u.) for E-cadherin (F) and β-catenin (K) in wt and MZ*vgll4a* embryos (t-test. $p= 0.0264$ for F and $p=0.0078$ in K). Scale bar: 10µm.

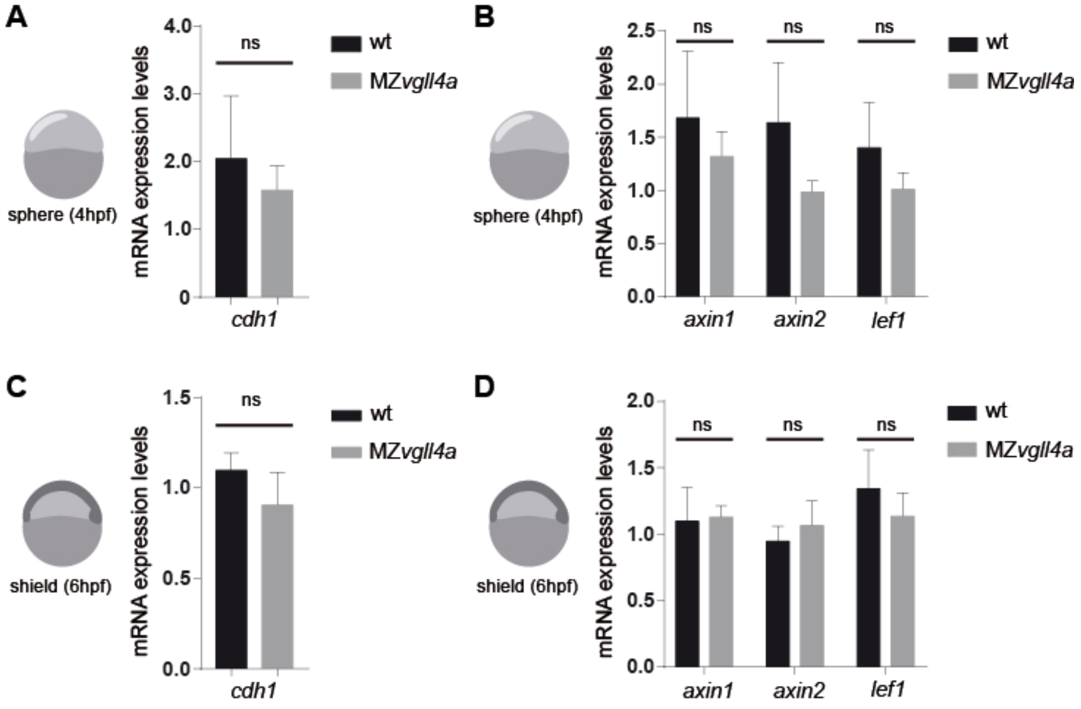

Supplementary Figure S8. Lack of vgll4a has no effect on the expression level of cdh1 and Wnt target genes. A-D) The graphs show the level of cdh1 and axin1, axin2 and lef1 (Wnt signaling targets) expression in wt and MZvgll4a embryos at sphere (A, B) and shield (C, D) stages. t-test. ns, not significant; A) p=0.4584; B) axin1 p= 0.3999, axin2 p=0.1204, lef1 p=0.2093; C) p=0.1786; D) axin1 p= 0.8609, axin2 p=0.4108, lef1 p=0.3575.

Supplementary Tables

Table S1. gRNAs used to mutate the different *vgll4* paralogues

Gene	gRNA
vgll4a	GCACCGCAAATCAAGAGTCA
Vgll4b	GGTACCTGCTGGCGCTCCAC
Vgll4l	GCCAGAGGCTCGTCCATGAC

Table S2. Primers and restriction enzymes used to genotype and determine mutagenesis.

Gene	Genotyping oligos		Restriction Enzyme
vgll4a	Fw	CCGACAAAGGACAAATGAACA	Hinf I
	Rv	GCAAATACATGATGGCTATGGA	
vgll4b	Fw	CGCTCGCACTGACCAAAA	BstX I
	Rv	ACACAGGACGCTGAACAATG	
vgll4l	Fw	AGAGAAAGGGCCCTCAGTAAGT	XcmI
	Rv	CACCTGTATTTGTGTTGTGGCT	

Table S3. List of qPCR primers used to detect transcript levels of the listed genes

Gene	Primer Fw	Primer Rv
eef1a1l1	TCTGTTACCTGGCAAAGGGG	GGAGTCGACGTGGCCAATAA
ccn1	GAGTGCAACTATGGGGCCAG	AGGTGCACTGGTGCTTACAG
arhgap18	CCGTACTGGCGGTTAAAGAG	GCCGCTGCACATTATACTGAC
axin1	AGCGTCTCAAGAGGGTTCG	AGGGACCGGTGGAGATGT
axin2	GAAGAAGAAGAGTGGAGTTGTAATGA	GGGTCACTGCGTCTGTAGG
lef1	ACGACACAGACCTGATGCAC	TTCTTGATGTGAGGTCTTTTGG
cdh1	GCCACAAGGTGTTTTCTGTG	CAGATCCACATCAGAGGAAGATT
